# Aging and self-reported health in 114 Latin American cities: gender and socio-economic inequalities

**DOI:** 10.1186/s12889-022-13752-2

**Published:** 2022-08-05

**Authors:** Marianela Castillo-Riquelme, Goro Yamada, Ana V. Diez Roux, Tania Alfaro, Sandra Flores-Alvarado, Tonatiuh Barrientos, Camila Teixeira Vaz, Andrés Trotta, Olga L. Sarmiento, Mariana Lazo

**Affiliations:** 1 Doctoral Program in Public Health, School of Public Health, Faculty of Medicine, University of Chile, Avenida Independencia, 939 Santiago, Chile; 2 Urban Health Collaborative, Dornsife School of Public Health, Drexel University, Philadelphia, PA USA; 3 Instituto Nacional de Salud Pública, Cuernavaca, Mexico; 4 Campus Centro-Oeste Dona Lindu, Federal University of São João del-Rei, Divinópolis, Brazil; 5 Institute of Collective Health, National University of Lanus, Buenos Aires, Argentina; 6 School of Medicine, Universidad de los Andes, Bogota, Colombia; 7 Department of Community Health and Prevention, Dornsife School of Public Health, Drexel University, Philadelphia, PA USA

**Keywords:** Self-reported health, Aging, Latin-America, Multilevel analysis, Urban health, Gender, Inequalities

## Abstract

**Background:**

Understanding how urban environments influence people’s health, especially as individuals age, can help identify ways to improve health in the rapidly urbanizing and rapidly aging populations.

**Objectives:**

To investigate the association between age and self-reported health (SRH) in adults living in Latin-American cities and whether gender and city-level socioeconomic characteristics modify this association.

**Methods:**

Cross-sectional analyses of 71,541 adults aged 25–97 years, from 114 cities in 6 countries (Argentina, Brazil, Colombia, Chile, El Salvador, and Guatemala), as part of the Salud Urbana en America Latina (SALURBAL) Project. We used individual-level age, gender, education, and self-reported health (SRH) data from harmonized health surveys. As proxies for socioeconomic environment we used a city-level socioeconomic index (SEI) calculated from census data, and gross domestic product (GDP) per-capita. Multilevel Poisson models with a robust variance were used to estimate relative risks (RR), with individuals nested in cities and binary SRH (poor SHR vs. good SRH) as the outcome. We examined effect modification by gender and city-level socioeconomic indicators.

**Results:**

Overall, 31.4% of the sample reported poor SRH. After adjusting for individual-level education, men had a lower risk of poor SRH (RR = 0.76; CI 0.73–0.78) compared to women, and gender modified the association between age and poor SRH (*p*-value of interaction < 0.001). In gender stratified models, the association between older age and poor SRH was more pronounced in men than in women, and in those aged 25–65 than among those 65+ (RR/10 years = 1.38 vs. 1.10 for men, and RR/10 years = 1.29 vs. 1.02 for women). Living in cities with higher SEI or higher GDP per-capita was associated with a lower risk of poor SRH. GDP per-capita modified the association between age (25–65) and SRH in men and women, with SEI the interaction was less clear.

**Conclusions:**

Across cities in Latin America, aging impact on health is significant among middle-aged adults, and among men. In both genders, cities with lower SEI or lower GDP per-capita were associated with poor SRH. More research is needed to better understand gender inequalities and how city socioeconomic environments, represented by different indicators, modify exposures and vulnerabilities associated with aging.

**Supplementary Information:**

The online version contains supplementary material available at 10.1186/s12889-022-13752-2.

## Background

By 2021, 56,6% of the global population lived in urban areas. Urbanization is especially high in Latin America, where the urban population is currently over 80%, together with North America among the highest across regions of the world [1, 2]. In addition to urbanization, an important global demographic trend is aging, with Latin America and the Caribbean experiencing relatively rapid increases in older populations [3]. Between 1990 and 2019, the percentage of the population aged 65 years and older almost doubled in Latin America and the Caribbean (from 6 to 11%) [3]. By 2050, countries in Latin America and Asia are expected to experience the greatest absolute growth in the share of the population above 65 years [4]. Worldwide, many older adults live in urban areas. For example, in 2015 in the Organization for Economic Cooperation and Development (OECD) region, the share of the older population (65+) living in cities was 43% with trends showing more rapid growth in metropolitan areas compared to non-metropolitan areas [5, 6]. Hence, understanding how urban environments influence people’s health across adulthood, and especially as individuals age, is an important research goal. Pursuing this goal can help identify ways to improve the health of older adults in the rapidly urbanizing and rapidly aging populations of Latin America and other similar regions.

Self-rated or self-reported health (SRH) has long been considered a reliable indicator of biological and mental health [7, 8], and a good predictor of morbidity and mortality [9, 10]. Mavaddat et al. for the UK context (study in 25,268 individuals aged 39–79) examined the strength of the association of single and multi-morbidity with SRH. They concluded that SRH provides a simple, integrative patient-centered assessment for evaluation of illness in the context of multiple chronic disease diagnoses [11].

SRH varies widely between [12–14] and within [15, 16] countries. A study that comprised 33 major metropolitan areas from eleven European countries reported that SRH was poorer in certain areas of the UK and Germany and better in areas of Sweden and Belgium [13]. Community and area socio-economic characteristics have also been linked to SRH within countries. For example, income inequality and poverty at the community or local area level, have been found to be associated with poor SRH in Chile [17], Colombia [18], United States [19] Wales [20] and Japan [21]. These findings suggest SRH can be influenced by place and living conditions.

Most studies on SRH differences have analyzed individuals within countries [22–24], with few studies conducted to understand how SRH varies across cities or is influenced by features of urban areas. The socioeconomic, physical and built environment features of cities can impact the access to resources that shape a healthy life such as good education, jobs, healthy diet, physical activity facilities, active transportation, etc. [25–27].

Loss of physical function, chronic pain, and the onset of chronic diseases result in worsening average SRH as populations age [22, 28]. Many studies analyzing the association between aging and SRH have focused on elderly populations [23, 29–31]. However, the aging process is a continuum that can be explored throughout adulthood [32]. Characterizing SRH across the entire age span of adulthood may help us identify key modifiable conditions to improve the aging process.

Furthermore, several cross-sectional studies have shown that women tend to have poorer SRH than men [33–36]. Several mechanisms could explain these gender differences including gender-based roles in leisure and personal activities [33], higher prevalence of chronic diseases among women [36], differences in how men and women assess their health [37] and differential access to education and employment [34] as well as differential health returns to education in women and men [38, 39].

To our knowledge, very limited evidence is available regarding the association between age and SRH in Latin America cities, and the extent to which these relationships vary by gender or are modified by social and economic features of cities. An existing review of 11 studies in Latin America suggests that features of the built and social environment (where two studies used neighborhood socioeconomic variables) are associated with SRH or health-related quality of life [40]. However, the focus of these studies was mostly on smaller neighborhoods within cities, and most of the studies were small (samples ranging from 685 to 2045 subjects), and comprised few countries (only Brazil, Colombia and Cuba were represented in the 11 studies) [40]. Studies examining gender differences in the region have been also limited in scope (population > 60 years) [35]. Based on capabilities and economic welfare models, socio-economic contexts of cities may impact SRH directly or may modify the aging effect on SRH (e.g. buffering or amplifying the impact) [41, 42]. However, the empirical evidence of these associations in cities in Latin America is lacking.

To fill these gaps, we examined the association between age and SRH across all cities of 100,000 residents or more in six Latin-American countries (Argentina, Brazil, Colombia, Chile, El Salvador, and Guatemala). We further evaluated if gender and city-level socioeconomic conditions are associated and/or modify the association between age and SRH. We used two main contextual social environment exposures: 1) a SALURBAL-derived Social Environment Index (SEI), which measures city-level material conditions of households (piped water, sewage system, overcrowding) and educational level which is a non-material household asset; and GDP per-capita, as a measure for cities SES, capturing dynamics of employment, growth, commerce, and business.

## Methods

### Study design and population

This is a cross-sectional, multi-level, multi-country study using data from the SALURBAL (Salud Urbana en América Latina) study. The SALURBAL study has integrated and harmonized health outcomes and physical and social environment data from 371 cities with more than 100,000 inhabitants in 11 Latin American countries [43]. The final analytical sample comprised 71,541 respondents, from 114 Latin-American cities in 6 countries **(**Additional file 1**)**. The average number of observations per city was 628, ranging from 16 to 3487.

Health outcomes and socio demographic information at the individual level were obtained from existing national surveys in each country and harmonized across countries using standardized definitions. For the current analyses, we used data for adults (25–97 years) from the following countries surveys (year of survey): Argentina (2013), Brazil (2013), Chile (2010), Colombia (2007), El Salvador (2004), and Guatemala (2002) (Additional file 2), as these were the only SALURBAL harmonized survey data with SRH data. Colombia (2007) included adults <= 69 years. Social environment data —inputs for SEI— was derived from the following countries censuses (year of census): Argentina (2010), Brazil (2010), Chile (2002), Colombia (2005), El Salvador (2007), and Guatemala (2002) (Additional file 2).

The SALURBAL study protocol was approved by the Drexel University Institutional Review Board (IRB) with ID #1612005035 and by appropriate site-specific IRBs.

### Outcome

SRH was measured on a 5-point Likert scale in each country. Although the question asked was fairly similar across countries, the response options differed. Adults were asked to answer the question “*In general, would you say your health is…”.* Argentina, Chile, Guatemala, and El Salvador used the exact same 5-point rating scale response including: “Poor”, “Fair”, “Good”, “Very Good” and “Excellent”. In Brazil and Colombia, the response rating scale included: “Very Poor”, “Poor”, “Fair”, “Good” and “Very Good”. During the data harmonization process, SRH was dichotomized into “Poor” (including “Very Poor”, “Poor”, “Fair”) and “Good” (including “Good”, “Very Good” and “Excellent”). This categorization facilitates comparison with other SRH epidemiological studies [19, 20, 29, 44–48].

Although SRH may be considered a more subjective measure of health compared to some objective measures of morbidity, we selected SRH as a proxy of global health status. SRH has shown to have high prognostic and predictive value of subsequent mortality and therefore represents a good proxy of overall health [10, 11, 49]. SRH captures health status more holistically and is a potent proxy of peoples’ health related quality of life. Indeed, improving SRH constitutes an important and common goal of health systems, and urban health.

### Exposures

Age (in years) was the primary individual-level exposure of interest and was obtained from the health surveys. Gender was our primary individual-level effect modifier, and it was inferred from self-reported sex from the survey responses (thus, we used sex as proxy for gender). We defined that if gender was an effect modifier, we would perform gender stratified analyses when investigating secondary effect modifiers.

The two co-primary city-level socioeconomic exposures included: Social Environment Index (SEI), and GDP per-capita.

The Social Environment Index, SEI, is a SALURBAL summary measure of city social development, which was already available at the time of the study [50]. SEI was created by combining four census-based indicators: education (% population with at least completed primary education among those aged 25 or above), water access (% households with access to piped water), sanitation (% households with access to a public sewage network) and reversed overcrowding (% households with more than 3 people per room). The index is the simple mean of these four city-level variables’ Z-scores. The four variables were selected because of their face validity in capturing different domains of the social environment and because of their predictive power of life expectancy differences across LATAM cities [50]. SEI has been used in previous research within the SALURBAL platform: for example, in relation to diabetes [51], hypertension [52] and green space [53]. Higher values of the city-SE index represent better city socioeconomic conditions.

GDP per-capita was obtained from a gridded global dataset [54], where subnational GDP estimates are based on an extensive econometric analysis [55] modeling GDP per capita for each city using a range of government, survey, and industry data. From this source annual gridded GDP (purchasing power parity in constant 2011 US dollars) per-capita, are available for the years from 1990 to 2015. To minimize de effect of annual fluctuations, we averaged 5 years, including the health survey year plus the 4 previous years.

Conceptually, these 2 city-level exposures can impact SRH directly or may modify the aging effect on SRH (e.g., buffering or amplifying the impact), thus we examined both the main effect of these exposures as well as their effect modification of the association between age and SRH.

### Covariates

Individual education was included as proxy for individual socioeconomic level as a covariate. This was categorized as: “Less than Primary Education”, “Primary Education Completed”, “High-School Completed”, and “University Completed or a Higher level”. City size (number of people in the city) as estimated for the survey year based on country projections was also used in descriptive analyses.

### Analysis

We used descriptive statistics to characterize the study population by outcome (Poor SRH and Good SRH), age categories (18–65 and 65 +), SEI and GDP per-capita tertiles. We used continuous age (in years) divided by ten, so that coefficients reflect differences in SRH associated with 10 years of age. To accommodate the non-linear association between poor SRH and age, we used a linear spline with a knot at 65 years. We selected the knot at 65 years of age (coinciding with the retirement age in most countries), based on descriptive analyses of the relation between age and SRH. Furthermore, since the association differed by gender, all analyses were gender stratified.

We conducted two-level Poisson regression models, with individuals nested within cities, and with robust variance estimation to account for misspecification of the variance by fitting count model for the binary outcome [56]. We used Poisson models to estimate relative risk (RR) in place of logistic models since our outcome was common. We first examined effect modification by gender, and then fitted the following gender stratified models. Model 1: included age and country; Model 2: included model 1 plus individual-level education; Model 3: included model 2 plus GDP per-capita tertiles; Model 4: model 2 plus city-SEI tertiles and Model 5: model 2 plus both GDP per-capita and city-SEI tertiles. Country was added as fixed effect in all models to account for unmeasured features of countries that may confound the association of interest, and to account for differences in surveys years. To examine whether city-level factors modified the association between age and poor SRH, we further constructed a model which included the interactions of SEI and GDP per-capita (separately) with age and present the results graphically in both relative and absolute scales using linear combination of coefficients and adjusted marginal predicted prevalences, respectively. These models adjusted for individual-level education and had country as fixed effect.

Associations and interactions were considered significant if the *p*-values was < 0.05. The analyses were performed using STATA 16.1 (StataCorp, College Station, TX).

## Results

The final analytical sample comprised 71,541 individuals, resident of 114 cities in 6 countries in Latin America. The mean age (SD) of the sample was 46.3 years (15.1) and 58% were women. Overall, 31% reported poor SRH. There were substantial variations in the proportion of poor SRH across the six countries. Those reporting poor SRH were older, more likely to be women and had lower educational attainment, as compared to those with good SRH. People reporting poor SRH were also different in terms of their city-level characteristics: they tended to live in cities with lower GDP per-capita, lower SEI, and smaller populations (Table 1).

**Table 1:** Characteristics of the study population by self-reported health status. SALURBAL Study (*n* = 71,541)

Characteristics	Poor SRH (*N* = 22,480)	Good SRH (*N* = 49,061)	Total (*N* = 71,541)	***p***-value*
***Country contribution to sample***				< 0.001
% Argentina	19.6	28.8	26.0	
% Brazil	48.8	44.4	45.8	
% Chile	4.6	2.8	3.4	
% Colombia	20.6	21.6	21.3	
% Guatemala & El Salvador	6.3	2.4	3.6	
***Individual-level sociodemographic characteristics***				
% Female	65.6	55.0	58.3	< 0.001
Mean (SD) Age in years	51.6 (15.5)	43.9 (14.2)	46.3 (15.1)	< 0.001
% 25–65 years	80.0	90.8	87.4	< 0.001
% > 65 years	20.0	9.2	12.6	
*Educational level*				
% Less than primary educ.	34.3	14.0	20.4	< 0.001
% Primary educ completed	33.9	27.6	29.6	
% High-School completed	24.8	38.8	34.4	
% University completed or higher level	7.0	19.5	15.6	
***City-level socioeconomic characteristics***				
Mean (SD) Socioeconomic Index, Z-score	−0.19 (0.97)	−0.02 (0.87)	0.17 (0.54)	< 0.001
Mean (SD) GDP per-capita	13,504.8 (8565.0)	14,769.6 (8937.9)	14,372.2 (8841.9)	< 0.001
Median GDP per-capita	10,401.6	11,225.4	11,225.4	
Mean (SD) Population size	3,549,314 (5027477)	3,754,636 (5330711)	3,690,119 (5238151)	
Median Population size	1,678,371	1,407,681	1,407,681	

*GDP* Gross Domestic Product, *HH* households, *SD* Standard deviation, *SRH* Self-rated health.

^*^ *P* values from Wilcoxon Mann Whitney test for continuous variables and Fisher exact test for categorical variables

Table 2 shows characteristics of the sample by age groups (18–65 and 65+). Compared to the younger group, older adults were more likely to be women (63% vs. 58%) and to report poor SRH (29% vs. 50%). Age distributions differed somewhat across countries. Argentina, Brazil, and Chile had a higher proportion in the older age group, while Colombia and Central America had larger proportion in the 18–65 age group. Older individuals had lower educational attainment but were more likely to live in cities with higher socioeconomic conditions as measured by SEI and GDP per-capita, and in cities with larger populations.

**Table 2:** Characteristics of the study population by age groups. SALURBAL Study (*N* = 71,541)

Variables	Age group 1 ***N*** = 62,556	Age group 2 ***N*** = 8985	Difference ***p***-value*
Age [range] in years	25–65	66–97	
***Country contribution to sample***			< 0.001
% Argentina	24.3	37.2	
% Brazil	45.6	47.1	
% Chile	3.1	5.2	
% Colombia	23.2	7.7	
% Guatemala & El Salvador	3.8	2.8	
***Individual-level sociodemographic characteristics***			
% Female	57.7	63.0	< 0.001
% Poor SRH	28.8	50.0	< 0.001
**Educational attainment**			
% Less than primary	16.9	44.7	< 0.001
% Primary Completed	29.7	28.9	
% High-School completed	37.0	16.2	
% University completed or higher level	16.4	10.1	
***City-level socioeconomic characteristics***			
Mean (SD) Socioeconomic Index, Z-score	−0.09 (0.92)	0.02 (0.78)	< 0.001
Mean (SD) GDP per-capita	14,080.12 (8782.2)	16,405.8 (8987.7)	< 0.001
Median GDP per-capita	11,225.4	16,263.2	
Mean (SD) Population size	3,601,395 (5139389)	4,307,837 (5843049)	< 0.001
Median Population size	1,407,681	1,646,057	

*GDP* Gross Domestic Product, *SD* Standard deviation, *SRH* Self-rated health.

^*^ *P* values from Wilcoxon Mann Whitney test for continuous variables and Chi-square test for categorical variables

Geographical differences in city-level socioeconomic contexts are presented in Additional file 3. In general, Chile showed the best living conditions (piped water, sewage system and overcrowding), adults’ education (completion of at least primary level) and GDP per-capita (mean and median). The lowest levels of SEI, GDP, education, and piped water are for Central America, who also bears the highest level of overcrowding (13.3%). Brazil is the country with the lower level of sewage system (58%) and the highest population (mean and median) per city.

Finally, the characteristics of the sample by city SEI and GDP per-capita tertile are shown in Additional file 4 and Additional file 5. Compared to tertiles 2 and 3 of SEI, individuals living in cities with lower social development (tertile 1) were younger, has a higher proportion of people aged 65+ and were less educated. They also had a higher proportion of people reporting poor SRH (38% in tertile 1 versus 29% in tertile 3). Also, cities in tertile 1 of SEI presented lower GDP per-capita and were smaller in terms of population (Additional file 4). Likewise, individuals living in cities with lower GDP per-capita (tertile 1) were slightly younger, had more people with less than primary education, and less people with university degree. They also had a higher proportion of people reporting poor SRH (36% versus 27% for tertile 3). Finally, cities in tertile 1 had the lower mean SEI and were of medium size population (Additional file 5).

In an initial model pooling both genders and adjusting for education (not shown in table), age was non-linearly associated with poor SRH (risk ratio [RR]/10 years of age and 95% CI, 1.24 CI, 1.22,1.27 for people aged 25–65 and RR = 0.99 CI, 0.96, 1.0 for people aged 65 or more). Men had lower risk of poor SRH (RR = 0.76 CI 0.73–0.78), than women. Furthermore, gender modified the association of age with SRH (*p* values < 0.001 for persons < 65 and < 0.001 for persons 65 and over) such that men showed stronger increases associated with age than women. Therefore, all subsequent analyses were stratified by gender.

Tables 3 and 4 show associations of age, education, city GDP and city SEI with poor SRH in women (*n* = 41,733) and men (*n* = 29,808), respectively. There was a positive association between age and poor SRH in women and men, and in both genders the associations were stronger among people aged 25–65 (RR = 1.29 95% CI, 1.26,1.32; RR = 1.38 CI, 1.35,1.42, for women and men, respectively), than among those over 65 years (RR = 1.02 CI, 0.99,1.06; RR = 1.10 95 CI, 1.06,1.15, for women and men, respectively). After adjusting for individual education and city-level socio-economic characteristics (models 2–5), age remained associated with poor SRH among those aged 25–65 years although the associations were slightly attenuated. In this age group, for each ten additional years the prevalence of reporting poor SRH increased 21% in women (RR = 1.21 CI, 1.18,1.24) and 30% in men (RR = 1.30 CI, 1.27,1.33). In contrast, age was not associated with poor SRH in women 65 years and above and was weakly associated with poor SRH in men 65 years or above (adjusted RR = 1.05 CI, 1.01,1.09). Model 3 for women and men shows that living in cities with less GDP per-capita (lowest tertile vs. the highest tertile) was associated with increased risk of poor SRH, even after adjusting for individual age and education level (RR = 1.24 CI 1.13,1.36; RR = 1.33 CI 1.18,1.49, for women and men respectively). Model 4 shows the adjusted association of tertiles of SEI and poor SRH, and demonstrates significant positive associations between cities with lower SEI (tertiles 1 and 2) and poor SRH compared to those with high SEI (tertile 3), in both men and women. To examine the independent association between SEI and GDP and SRH, we used a model that incorporates both factors (Model 5) and found that although there was some attenuation in the associations, these city-level variables remain significantly associated with poor SRH, for both men and women.

**Table 3:** Associations of age, education and city GDP with poor self-reported health in 114 cities in Latin America among women (*n* = 41,733)

Variable	Model 1: Age		Model 2: Age, adjusting for individual education		Model 3: Model 2 + GDP Tertiles		Model 4: Model 2 + SEI Tertiles		Model 5: Model 2 + GDP tertiles + SEI Tertiles	
	RR (95% CI)	***p***-value	RR (95% CI)	***p***-value	RR (95% CI)	***p***-value	RR (95% CI)	***p***-value	RR (95% CI)	***p***-value
**Individual-level factors**										
Age, per 10 years increase, among people aged 25–65 years	1.29 (1.26, 1.32)	< 0.001	1.21 (1.18, 1.24)	< 0.001	1.21 (1.18, 1.24)	< 0.001	1.21 (1.18, 1.24)	< 0.001	1.21 (1.18, 1.24)	< 0.001
Age, per 10 years increase, among people aged > 65 years	1.02 (0.99, 1.06)	0.19	0.97 (0.94, 1.00)	0.09	0.97 (0.94, 1.01)	0.09	0.97 (0.94, 1.01)	0.096	0.97 (0.94, 1.01)	0.096
Education less than primary			2.77 (2.59, 2.96)	< 0.001	2.76 (2.58, 2.96)	< 0.001	2.76 (2.58, 2.95)	< 0.001	2.76 (2.58, 2.95)	< 0.001
Education primary			2.39 (2.22, 2.57)	< 0.001	2.39 (2.22, 2.57)	< 0.001	2.39 (2.22, 2.57)	< 0.001	2.39 (2.22, 2.57)	< 0.001
Education secondary			1.60 (1.50, 1.71)	< 0.001	1.60 (1.50, 1.71)	< 0.001	1.60 (1.50, 1.71)	< 0.001	1.60 (1.50, 1.71)	< 0.001
Education university or higher			1.00 (reference)		1.00 (reference)		1.00 (reference)		1.00 (reference)	
**City-GDP per capita and SEI**										
GDP per capita: tertile 1					1.24 (1.13, 1.36)	< 0.001			1.19 (1.13, 1.36)	0.005
GDP per capita: tertile 2					1.07 (0.98, 1.17)	0.15			1.06 (0.97, 1.16)	0.208
GDP per capita: tertile 3					1.00 (reference)				1.00 (reference)	
SEI: tertile 1							1.29 (1.17, 1.42)	< 0.001	1.23 (1.11, 1.37)	< 0.001
SEI: tertile 1							1.15 (1.04, 1.26)	0.004	1.17 (1.07, 1.19)	0.001
SEI: tertile 1							1.00 (reference)		1.00 (reference)	

All models consider country as fixed effect

**Table 4:** Associations of age, education, and city GDP with poor self-reported health in cities in 114 cities in Latin-America among men (*n* = 29,808)

Variable	Model 1: Age		Model 2: Age, adjusting for individual education		Model 3: Model 2 + GDP Tertiles		Model 4: Model 2 + SEI Tertiles		Model 5: Model 2 + GDP & SEI Tertiles	
	RR (95% CI)	***p***-value	RR (95% CI)	***p***-value	RR (95% CI)	***p***-value	RR (95% CI)	***p***-value	RR (95% CI)	***p***-value
**Individual-level factors**										
Age, per 10 years increase, among people aged 25–65 years	1.38 (1.35, 1.42)	< 0.001	1.30 (1.27, 1.33)	< 0.001	1.30 (1.27, 1.33)	< 0.001	1.30 (1.27, 1.33)	< 0.001	1.30 (1.27, 1.33)	< 0.001
Age, per 10 years increase, among people aged > 65 years	1.10 (1.06, 1.15)	< 0.001	1.05 (1.01, 1.09)	0.02	1.05 (1.01, 1.09)	0.02	1.05 (1.01, 1.09)	0.021	1.05 (1.01, 1.09)	0.022
Education: less than primary			3.20 (2.89, 3.55)	< 0.001	3.20 (2.89, 3.55)	< 0.001	3.19 (2.88, 3.55)	< 0.001	3.19 (2.88, 3.55)	< 0.001
Education: primary			2.64 (2.39, 2.92)	< 0.001	2.64 (2.39, 2.92)	< 0.001	2.64 (2.39, 2.91)	< 0.001	2.64 (2.39, 2.91)	< 0.001
Education: secondary			1.81 (1.64, 1.99)	< 0.001	1.81 (1.64, 1.99)	< 0.001	1.81 (1.64, 1.99)	< 0.001	1.81 (1.64, 1.99)	< 0.001
Education university or higher			1.00 (reference)		1.00 (reference)		1.00 (reference)		1.00 (reference)	
**City-GDP per capita and SEI**										
GDP per capita: tertile 1					1.33 (1.18, 1.49)	< 0.001			1.27 (1.11, 1.46)	0.001
GDP per capita: tertile 2					1.09 (0.98, 1.22)	0.11			1.09 (0.98, 1.21)	0.117
GDP per capita: tertile 3					1.00 (reference)				1.00 (reference)	
SEI: tertile 1							1.39 (1.24, 1.56)	< 0.001	1.30 (1.17, 1.45)	< 0.001
SEI: tertile 1							1.21 (1.07, 1.36)	0.002	1.24 (1.11, 1.39)	< 0.001
SEI: tertile 1							1.00 (reference)		1.00 (reference)	

All models consider country as fixed effect

**Fig. 1:**
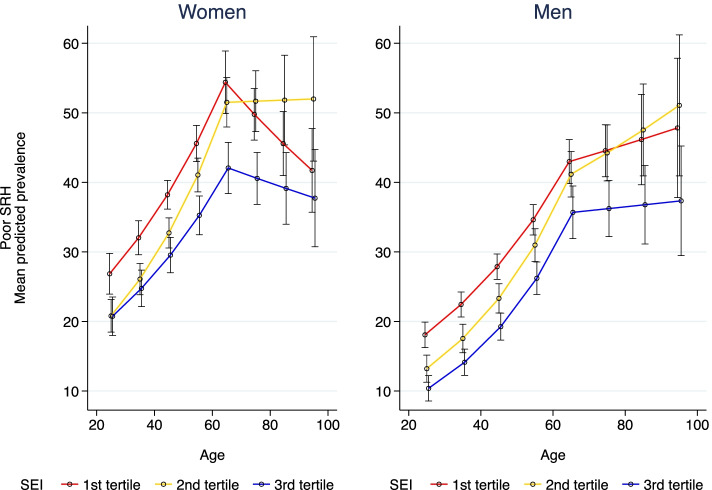
Prevalence of poor SRH by age, SEI tertile and gender. SALURBAL Study (*n* = 71,541)

**Fig. 2:**
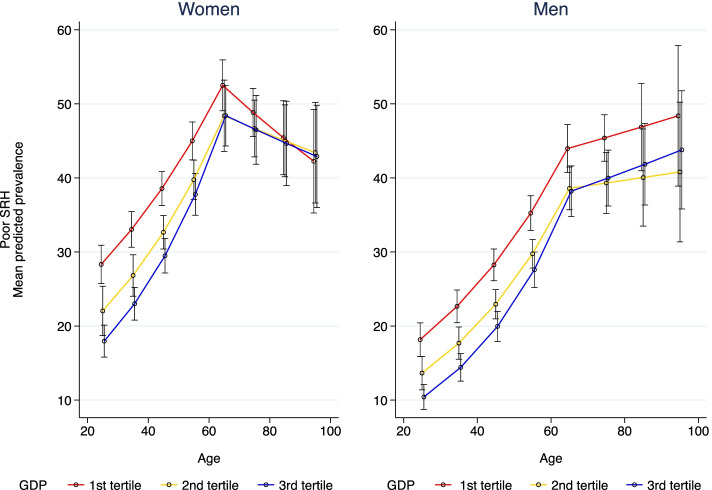
Prevalence of poor SRH by age, GDP tertiles and gender. SALURBAL Study (*n* = 71,541)

Results examining the role of SEI and GPD per-capita as effect modifiers are shown in Figs. 1, 2 respectively. Figure 1 shows adjusted marginal prevalences of SRH by age and SEI in each gender derived from gender specific models including interactions between age and SEI, and adjusting for individual education. We observed a more significant interaction for men in the younger group, with slightly stronger impact of increasing age on SRH among those living in cities with medium SEI (tertile 2).

Figure 2 for GDP shows, that among those aged 25–65 years, the association of age with poor SRH was stronger at higher than at lower levels of city GDP (*p*-value for interaction between age and GDP per-capita =0.001 for women and < 0.001 for men). For men and women aged 65 and older no interaction between age and GDP was observed. Additional files 6 and 7, shows that results of the interaction model in the relative scale for SEI and GDP, respectively, are consistent with the results on the absolute scale.

## Discussion

Based on cross-sectional health surveys from six Latin-American countries, we found that higher age was associated with poor SRH in men and women. In both genders the association between aging and poor SRH was stronger in middle-aged adults (25–65 years), than in older adults (65+) and remained significant even after accounting for individual level education and city-level socio economic characteristics. We further found that associations of age with poor SRH were stronger in men than in women. In both genders, both lower levels of city SEI and GDP per-capita were associated with poor SRH, even after adjusting for each other, with somewhat stronger associations for SEI as compared to GDP per capita. In addition, we found that GPD per-capita modified the associations between age and poor SRH, such that in middle-aged adults the age-SRH associations were stronger in cities with higher GPD per-capita. This interaction was less clear for city-SEI.

In our sample, after adjusting for education, the prevalence of poor SRH was 31.6% higher in women than in men. These findings are in line with previous studies showing that women are more likely to report poorer SRH levels than men [29–32]. The postulated mechanisms behind these gender differences include differential exposure to factors that increase the risk of adverse health outcomes and differential vulnerability to these risk factors [57]. Some of the reasons for lower SRH in Latin America women could also lie in culture and social structure. Women bear most of household chores, have fewer job opportunities and receive less income [35]. Women represent a high proportion of workers in the informal sector in Latin America. Informality, which exposes women to unsafe conditions including risk of sexual harassment, often leaves women without the protection of labor laws, social benefits, health insurance or paid sick leave [58]. Further research is needed to better understand gender gaps in SRH in Latin America across adulthood.

Our results show that the impact of aging on SRH begins in early to mid-adulthood, we found stronger associations of higher age with poor SRH among young and middle-aged adults (25–65) than in older adults (65–97). Most previous studies analyzed the age effects on SRH focus on older population [22, 23, 29–31], and there are no comparable studies that explored age as main exposure for SRH across all adulthood. Additionally, cross-country comparisons in SRH cover mainly European or developed countries [12–14]. To our knowledge, there are two smaller studies, one from Giron, Spain and another one in Canada, with similar findings [45, 59]. The lack of association among older adults may be due to survival or selection bias, as people with worse health have higher mortality and those in poor health are unlikely to respond to surveys [22]. In our sample men 65+ showed a slight positive association of age with poor SRH, but this association was considerably less than that found in men aged 25–65 years. Birth cohort effects could also play a role in the associations of age with SRH that we report.

We also found that the association with age seemed to be stronger in men than in women. Reasons for these findings should be further studied in the region. A study from 2000, reported gender differences in SRH assessments in the elderly [37], noting that women tend to consider a broader range of information (beyond serious illnesses) when rating their health, while men would focus more on life threatening conditions [37], thus making SRH a strong mortality predictor for men only [37]. However, Zajacova et al. in a more recent (2017) and larger study for US individuals aged 25–84, concluded that SRH has similar meaning for men and women, and that both groups use a wide range of health-related information in forming their health judgements [60]. They found that women report worse SRH than men but only until mid-adulthood; the gender difference was reversed at older ages. They further reported that the excess of poor SRH among women disappears when differences in socio-economic and health covariates were considered [60]. Rohlfsen & Kronenfeld, using longitudinal data for US population (mean age 56; SD 3 years), reported a significantly faster decline overtime in SRH in men as compared to women [57]. Transition to retirement, smoking status, and onset of chronic conditions could explain this faster decline of SRH in men. In women, lower baseline SRH was related to employment status and socioeconomic level; however, after adjudgments for structural and health status factors, women reported better SHR than men [57]. A qualitative study in 62 Wisconsin adults concluded that the way people take into account health issues to formulate their answers to the SRH question varies by socioeconomic status, gender and age [61]. An additional challenge in comparing age associations in men and in women using RR as we did is that the levels of poor SRH differ by gender being significantly higher in women than in men. Therefore, a smaller RR in women associated with age is compatible with a similar or even larger absolute difference (as suggested by Figs. 1 and 2). Further work is needed to better understand the differences in the associations of age with SRH in men and women and the extent to which they reflect differences in measurement, in exposures or in vulnerabilities by gender.

After adjusting for individual education, we found an independent association between both city-level SEI and GDP per-capita with SRH, in both men and women. These results suggest that these two social economic constructs have different mechanisms by which they impact SRH. SEI can exert a more direct influence of SRH as the index comprises crucial household assets (clean water, sewage system, dwelling space, and education), needed to accomplish basic levels of health and wellbeing, while GDP per-capita representing wealth, constitutes cities potential to achieve better health in a paradigm of economic welfare, perhaps, in a more indirect way. As stated in the introduction SEI approaches follow better (though not perfectly) the capabilities approach while GDP per-capita constitutes the classical welfarist approach to health and healthcare [41, 42]. One study at country level, found that the aggregated effect of education was twice the effect of income on cognitive functioning in adult aged 50 years and older, and thus indirectly moderated the effect of income on cognitive functioning. They further report that the effect sizes varied strongly between countries, concluding that country’s GDP per-capita seems to influence cognitive functioning [62].

Other studies examining how urban contexts impact SRH have focused on neighborhood level factors [40, 63]. Within a review for Latin American countries, two out of 11 studies showed positive associations of neighborhoods socioeconomic levels and SRH, while others four showed that built environment features (parks, walkability, lack of noise) which are known to encourage healthy lifestyle, were associated to better SRH [40]. We did not explore built environment characteristics of the cities and therefore future research should widen the dimensions (and variables) in which the urban settings can affect and modify the age-SRH association in Latin America.

Finally, we found that GDP per-capita (more than SEI) modified the association of age and SRH in men and women (25–65 years). Among people aged 25–65 we found stronger associations of age with SRH in cities with higher rather than lower GDP. The potential mechanisms for these results may include larger inequalities among cities with higher GDP per-capita and construct bias regarding self-rated health [12]. Which seems consistent with a recent study that used GDP per-capita at country level, reporting socioeconomic inequalities in physical and cognitive functions in a large sample (37 cohorts from 28 countries). The authors conclude that such inequalities exist across different social contexts, with varying magnitudes and appear to be larger in higher-income countries [64]. Further work is needed to understand how city GDP modifies exposures and vulnerabilities associated with aging.

### Limitations and strengths

Our research has some limitations. Firstly, SRH may be considered a subjective measure of health compared to some objective measures of morbidity. Furthermore, to control for SES at the individual level, we relied only on educational level as a proxy for individual SES. Secondly, the cross-sectional nature of the study limits our ability to make causal inferences. Thirdly, although we had few missing data (4.3% - see Additional file 1), country responders may not be representative of all health status and age strata within included cities. The potential underrepresentation of elderly people, and people with severe conditions or multimorbidity might introduce some bias towards good SRH. Moreover, one of the country surveys (Colombia) only included people under 70 years of age.

We also encountered lack of alignment in the calendar-years of the data sources, e.g. year of the survey (source of SRH and individual covariates) and the census year (source of the SEI). The gap between survey data and census was largest for Chile (8-year difference), and smallest for Colombia, Argentina, Brazil and El Salvador (2–3 year difference). Although there may have been differences in the prevalence of poor SRH over time, we do not anticipate differences in the associations that were examined in this study. Another related limitation is the fact that Guatemala data is the oldest (2002), but similarly, we do not expect changes in the associations over time. Finally, the role of built environment characteristics of the cities (such as air pollution, greenness, climate, or traffic) on SRH, was beyond the current scope of this manuscript. Future studies in SALURBAL are very well position to address this research question.

Despite these shortcomings, this study has important strengths. This is one of the largest studies to date, with a harmonized dataset, focusing on a wide and heterogeneous range of Latin American cities (114 cities from 6 countries), most of the countries are classified in the category of middle-income by the World Bank. Our sample includes adults with a wide range of ages and thus extends our knowledge of SRH across adulthood. Finally, the study multi-level structure, nesting people within cities, allows accounting for unmeasured city contextual factors which may have a role in shaping SRH as people age.

### Conclusions

In Latin American cities aging was associated with higher risk of poor SRH, but this association was stronger in adults from 25 to 65 than in those over 65. Although women are more likely to report poor SRH, we found that the association between aging and poor SRH was more pronounced among men. City SEI and GDP per-capita were associated with SRH with stronger and most consistent effects for SEI. GDP per-capita modified the association between age and poor SRH such that high GDP was associated with stronger age association.

The main policy and research implications are the need to better understand the gender differences in the experience of perceived health with aging in Latin American cities. Women rate their health worse than men, but men feel a stronger deterioration in SRH as they age. Future research could explore the extent to which gender-based cultural, social, or political issues shape these trajectories in Latin American cities. In addition, more research (qualitative and quantitative) is needed to better understand how city socioeconomic environments represented by different indicators (such material resources vs. GDP per-capita) exert potentially independent influences on the age effect on SRH, suggesting different mechanisms behind these associations. Studies should also investigate what Latin American people consider when rating their health. On the other hand, realizing that the age effect on SRH is stronger in younger adults rather than people over 65 years is a key finding that should guide the design of better policies that support healthy adulthood in urban settings.

Additionally, actions to improve (perceived) health status should begin at early ages in adulthood considering the different gender trajectories as people age and the role of cities in delaying and attenuating the impact of age in SRH in Latin America countries.

## Supplementary Information

**Additional file 1.** Derivation of the analytical sample. SALURBAL Study

**Additional file 2.** Data sources per country and analytical sample (individuals and cities)

**Additional file 3.** Socioeconomic characteristics of the cities in the study, by country

**Additional file 4. **Characteristics of the study population by SEI tertiles. SALURBAL Study (*N* = 71,541)

**Additional file 5. **Characteristics of the study population by GDP per-capita (in constant 2011 USD) tertiles. SALURBAL Study (*N* = 71,541)

**Additional file 6.** Association between individual age and poor self-reported health by tertiles of Socioeconomic Index, age, and gender.

**Additional file 7.** Association between individual age and poor self-reported health by tertiles of GDP, age, and gender

## Data Availability

The SALURBAL project welcomes queries from anyone interested in learning more about its dataset and potential access to data. To learn more about SALURBAL visitbhttps://Drexel.edu/lac/ or contact the project at salurbal@drexel.edu.

## Abbreviations

GDP per-capita: Gross Domestic Product per-capita
Good SRH: good or better self-reported or self-rated health
HRQoL: Health-related Quality-of-Life
IRB: Institutional Review Board
LATAM: Latin American
Poor SRH: Poor self-rated health (includes the categories of fair, poor and very poor self-reported or self-rated health)
SALURBAL: Salud Urbana en América Latina
SEI: Socioeconomic Index
SRH: Self-reported or self-rated health
