# Supplementary figures and images for: Aging and self-reported health in 114 Latin American cities: gender and socio-economic inequalities
Source: BMC Public Health. 2022 Aug 5;22:1499. doi: 10.1186/s12889-022-13752-2 (PMC9356475; doi:10.1186/s12889-022-13752-2)

**Additional file 1: Derivation of the analytical sample. SALURBAL Study**

**
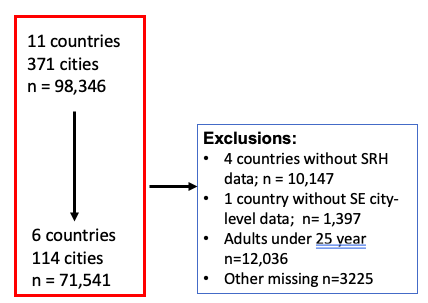
**

Supplement: Supplementary file 1 — Additional file 1. Derivation of the analytical sample. SALURBAL Study [file 12889_2022_13752_MOESM1_ESM.docx]
