# Supplementary material for: Aging and self-reported health in 114 Latin American cities: gender and socio-economic inequalities
Source: BMC Public Health. 2022 Aug 5;22:1499. doi: 10.1186/s12889-022-13752-2 (PMC9356475; doi:10.1186/s12889-022-13752-2)
Supplement: Supplementary file 2 — Additional file 2. Data sources per country and analytical sample (individuals and cities) [file 12889_2022_13752_MOESM2_ESM.docx]

**Additional file 2: Data sources per country and analytical sample (individuals and cities)**

| Country | Survey year | Census year | Final sample | Number of cities |
| --- | --- | --- | --- | --- |
| Argentina | 2013 | 2010 | 18,574 | 33 |
| Brazil | 2013 | 2010 | 32,743 | 27 |
| Chile | 2010 | 2002 | 2,398 | 19 |
| Colombia | 2007 | 2005 | 15,221 | 33 |
| El Salvador | 2004 | 2007 | 1,479 | 1 |
| Guatemala | 2002 | 2002 | 1,126 | 1 |
| ***Total*** |  |  | **71,541** | **114** |
