# Supplementary material for: Aging and self-reported health in 114 Latin American cities: gender and socio-economic inequalities
Source: BMC Public Health. 2022 Aug 5;22:1499. doi: 10.1186/s12889-022-13752-2 (PMC9356475; doi:10.1186/s12889-022-13752-2)
Supplement: Supplementary file 3 — Additional file 3. Socioeconomic characteristics of the cities in the study, by country [file 12889_2022_13752_MOESM3_ESM.docx]

**Additional file 3: Socioeconomic characteristics of the cities in the study, by country**

| Country | Argentina | Brazil | Chile | Colombia | Central America |
| --- | --- | --- | --- | --- | --- |
| Socioeconomic index (SEI) | 0.34 (0.27) | 0.02 (0.49) | 0.93 (0.17) | 0.34 (0.27) | -0.62 (0.37) |
| Crude SEI components |  |  |  |  |  |
| - % Of adults (>25) that completed at least primary education | 79.94 (2.30) | 70.69 (3.48) | 85.26 (3.51) | 78.03 (5.54) | 67.73 (2.48) |
| - % Of HH with piped water | 98.72 (1.03) | 96.84 (4.69) | 98.98 (1.45) | 96.80 (4.60) | 89.02 (2.97) |
| - % Of HH with sewage system from a public network | 65.53 (15.3) | 58.24 (24.9) | 95.95 (3.22) | 86.01 (16.5) | 73.77 (3.34) |
| - % Of HH living in overcrowding conditions (<3 people per room) | 3.97 (1.26) | 4.54 (3.26) | 3.88 (1.32) | 5.63 (3.37) | 13.31 (3.37) |
| Mean (SD) GDP per capita | 20774.0 (9823.9) | 13188.9 (6993.8 | 22890.2 (14393) | 8521.8 (2536.1) | 9941.2 (4437.5) |
| Median GDP per-capita | 20249 | 11225.4 | 22047.6 | 8767.2 | 6070 |
| Mean Population projection | 3336161 (5767449) | 4762502  (5856887) | 2048049  (2651768) | 2319925 (2851024) | 2252403 (619067) |
| Median population | 549768 | 3019450 | 349712 | 772862 | 1712346 |
| GDP= Gross Domestic Product; HH= household; SD= Standard deviation | | | | | |
