# Supplementary material for: Aging and self-reported health in 114 Latin American cities: gender and socio-economic inequalities
Source: BMC Public Health. 2022 Aug 5;22:1499. doi: 10.1186/s12889-022-13752-2 (PMC9356475; doi:10.1186/s12889-022-13752-2)
Supplement: Supplementary file 4 — Additional file 4. Characteristics of the study population by SEI tertiles. SALURBAL Study (N = 71,541) [file 12889_2022_13752_MOESM4_ESM.docx]

**Additional File 4: Characteristics of the study population by SEI tertiles. SALURBAL Study (N=71,541)**

| Characteristics | SEI Tertile 1 | SEI Tertile 2 | SEI Tertile 3 | p-value |
| --- | --- | --- | --- | --- |
| SEI range | (-3.4) – (- 0.2) | (-0.2) - (0.40) | (0.4) – (1.5) |  |
| **Individual-level sociodemographic characteristics** | | | | |
| Mean (SD) Age in years | 44.8 (14.4) | 47.6 (15.6) | 46.5 (14.8) | <0.001 |
| % 25-65 years old | 90 | 85 | 88 | <0.001 |
| % >65 years old | 10 | 15 | 12 |  |
| % Female | 59 | 58 | 58 | 0.01 |
| % Poor SRH | 37.6 | 28.5 | 28.1 | <0.001 |
| **Educational attainment** | | |  | <0.001 |
| % Less than primary | 24.3 | 18.7 | 18.0 |  |
| % Primary Completed | 27.3 | 28.8 | 33.1 |  |
| % High-School completed | 34.5 | 34.1 | 34.8 |  |
| % University completed or higher level | 13.9 | 18.4 | 14.1 |  |
| **Country contribution to the sample** | | | | <0.001 |
| % Argentina | 14.1 | 38.8 | 23.6 |  |
| % Brazil | 58.8 | 46.6 | 30.3 |  |
| % Chile | - | 0.1 | 11.1 |  |
| % Colombia | 16.2 | 14.6 | 35.0 |  |
| % Guatemala & El Salvador | 10.9 | - | - |  |
| **Other city-level socioeconomic characteristics (Z-score)** | | | | |
| Mean (SD) GDP per capita | -0.54 (0.33) | 0.45 (0.67) | 0.02 (1.06) | <0.001 |
| Mean (SD) Pop projection | 0.01 (0.39) | 1.87 (2.64) | 0.55 (0.94) | <0.001 |
| ^*^ P values from ANOVA global test for continuous variables and Chi-square test for categorical variables  GDP= Gross Domestic Product; SD= Standard deviation; SRH= Self-rated health | | | | |
