# Supplementary material for: Aging and self-reported health in 114 Latin American cities: gender and socio-economic inequalities
Source: BMC Public Health. 2022 Aug 5;22:1499. doi: 10.1186/s12889-022-13752-2 (PMC9356475; doi:10.1186/s12889-022-13752-2)
Supplement: Supplementary file 5 — Additional file 5. Characteristics of the study population by GDP per-capita (in constant 2011 USD) tertiles. SALURBAL Study (N = 71,541) [file 12889_2022_13752_MOESM5_ESM.docx]

**Additional File 5: Characteristics of the study population by GDP per-capita (in constant 2011 USD) tertiles. SALURBAL Study (N=71,541)**

| **Characteristics** | **Tertile 1** | **Tertile 2** | **Tertile 3** | **p-value *** |
| --- | --- | --- | --- | --- |
| GDP per capita, USD [range] | [$2144 – $9093] | [$9260-$18723] | [$19209- $64667] |  |
| **Individual-level sociodemographic characteristics** | | | | |
| Mean (SD) Age in years | 44.7 (14) | 46.2 (15) | 48.2 (16) |  |
| % 25-65 years old | 91.2 | 87.4 | 83.2 | <0.001 |
| % >65 years old | 8.8 | 12.6 | 16.8 |  |
| % Female | 58.9 | 57.9 | 58.1 | 0.052 |
| % Poor SRH | 35.6 | 30.7 | 27.4 | <0.001 |
| **Educational attainment** | | |  | <0.001 |
| % Less than primary | 23.0 | 21.0 | 16.9 |  |
| % Primary Completed | 28.4 | 31.5 | 29.1 |  |
| % High-School completed | 35.1 | 33.4 | 34.7 |  |
| % University completed or higher level | 13.6 | 14.1 | 19.2 |  |
| **Country contribution to the sample** | | | | <0.001 |
| % Argentina | 4.0 | 30.9 | 45.8 |  |
| % Brazil | 47.8 | 40.3 | 48.6 |  |
| % Chile | 0.4 | 4.5 | 5.6 |  |
| % Colombia | 42.2 | 19.1 | - |  |
| % Guatemala & El Salvador | 5.7 | 5.1 | - |  |
| **Other city-level socioeconomic characteristics (Z-score)** | | | | |
| Mean (SD) Socioeconomic Index | -0.36 (1.03) | -0.15 (1.00) | 0.32 (.35) | <0.001 |
| Mean (SD) Population projection | 0.35 (0.82) | 0.07 (0.55) | 2.15 (2.68) | <0.001 |
| ^*^ P values from ANOVA global test for continuous variables and Chi-square test for categorical variables  GDP= Gross Domestic Product; SD= Standard deviation; SRH= Self-rated health | | | | |
