# Supplementary material for: Aging and self-reported health in 114 Latin American cities: gender and socio-economic inequalities
Source: BMC Public Health. 2022 Aug 5;22:1499. doi: 10.1186/s12889-022-13752-2 (PMC9356475; doi:10.1186/s12889-022-13752-2)
Supplement: Supplementary file 6 — Additional file 6. Association between individual age and poor self-reported health by tertiles of Socioeconomic Index, age, and gender. [file 12889_2022_13752_MOESM6_ESM.docx]

**Additional Figure 6: Adjusted* association between individual age and poor self-reported health by tertiles of Socioeconomic Index, age, and gender.**

*P-value* interactions

Women: age <65 *p-value* = 0.08; age 65+ *p-value* = 0.07

Men: age <65 *p-value* = 0.002; age 65+ *p-value* = 0.51

*Model adjusted for individual level education, and country (as fixed effect)
